# Cancer Cells Can Exhibit a Sparing FLASH Effect at Low Doses Under Normoxic *In Vitro-*Conditions

**DOI:** 10.3389/fonc.2021.686142

**Published:** 2021-07-29

**Authors:** Gabriel Adrian, Elise Konradsson, Sarah Beyer, Anders Wittrup, Karl T. Butterworth, Stephen J. McMahon, Mihaela Ghita, Kristoffer Petersson, Crister Ceberg

**Affiliations:** ^1^Division of Oncology and Pathology, Clinical Sciences, Skåne University Hospital, Lund University, Lund, Sweden; ^2^Department of Hematology, Oncology and Radiation Physics, Skåne University Hospital, Lund, Sweden; ^3^Department of Medical Radiation Physics, Clinical Sciences, Lund University, Lund, Sweden; ^4^Wallenberg Center for Molecular Medicine, Lund, Sweden; ^5^Patrick G. Johnston Centre for Cancer Research, Queen’s University Belfast, Belfast, United Kingdom; ^6^Medical Research Council Oxford Institute for Radiation Oncology, Department of Oncology, University of Oxford, Oxford, United Kingdom

**Keywords:** FLASH, ultra-high dose rate irradiation, clonogenic assay, normoxia, radiotherapy, radiobiology, radioresistance, cancer cell lines

## Abstract

**Background:**

Irradiation with ultra-high dose rate (FLASH) has been shown to spare normal tissue without hampering tumor control in several *in vivo* studies. Few cell lines have been investigated *in vitro*, and previous results are inconsistent. Assuming that oxygen depletion accounts for the FLASH sparing effect, no sparing should appear for cells irradiated with low doses in normoxia.

**Methods:**

Seven cancer cell lines (MDA-MB-231, MCF7, WiDr, LU-HNSCC4, HeLa [early passage and subclone]) and normal lung fibroblasts (MRC-5) were irradiated with doses ranging from 0 to 12 Gy using FLASH (≥800 Gy/s) or conventional dose rates (CONV, 14 Gy/min), with a 10 MeV electron beam from a clinical linear accelerator. Surviving fraction (SF) was determined with clonogenic assays. Three cell lines were further studied for radiation-induced DNA-damage foci using a 53BP1-marker and for cell cycle synchronization after irradiation.

**Results:**

A tendency of increased survival following FLASH compared with CONV was suggested for all cell lines, with significant differences for 4/7 cell lines. The magnitude of the FLASH-sparing expressed as a dose-modifying factor at SF=0.1 was around 1.1 for 6/7 cell lines and around 1.3 for the HeLa_subclone_. Similar cell cycle distributions and 53BP1-foci numbers were found comparing FLASH to CONV.

**Conclusion:**

We have found a FLASH effect appearing at low doses under normoxic conditions for several cell lines *in vitro*. The magnitude of the FLASH effect differed between the cell lines, suggesting inherited biological susceptibilities for FLASH irradiation.

## Introduction

The FLASH effect denotes the radiobiological phenomenon that a given absorbed dose of ionizing radiation produces less damage at ultra-high dose rates (>40-100 Gy/s), as compared to the lower dose rates conventionally used in radiotherapy (CONV, about 0.1 Gy/s). Experimental evidence for the FLASH effect has been demonstrated *in vivo* in various preclinical settings (1–5), as well as in one first-in-human case of a patient with multi-resistant cutaneous lymphoma (6).

Although there is no proven mechanistic explanation for the FLASH effect, the limited *in vivo* data available suggest that more sparing may occur in physoxic normal tissues than in severely hypoxic or nearly anoxic tumors (7). One plausible theory describes the FLASH effect as a protective, radiation-induced hypoxia, tentatively explained by the so-called transient oxygen depletion (TOD) hypothesis as a net effect of radiolytic oxygen consumption exceeding the physiologic supply (8–10). According to the TOD hypothesis, the degree of sparing would be largest for already hypoxic tissues, where further oxygen depletion can be substantial. No effect would be expected at normoxia, where radiolytic oxygen consumption would not be sufficient for producing hypoxic radioresistance, or at anoxia where there can be no further oxygen depletion. However, recent work by Labarbe et al. has indicated, based on simulations and mathematical modelling, that the TOD hypothesis is most likely not sufficient to account for the FLASH effect reported at dose levels limited by normal tissue toxicity (11). Consequently, the authors suggest that other mechanisms may regulate the process and that a FLASH effect may be present also at normoxic conditions, even at relatively low dose levels.

Surprisingly, few recent *in vitro* studies with clonogenic assays have been reported to support these basic assumptions, and the limited data available suggests that different cell lines may have different susceptibility to the FLASH effect. In previous work, we studied clonogenic survival of the human prostate cancer DU145 cell line and found a FLASH effect at lower oxygen concentrations but no significant differences in normoxic conditions (12). Montay-Gruel et al. studied the murine glioblastoma H454 cell line and demonstrated significant FLASH effects both at 4% oxygen concentration and in normoxic conditions (13). For normal human lung fibroblasts (14) and lung cancer A549 (15), no difference in survival at different dose-rates in normoxia was reported. Neither has the use of laser-accelerated protons revealed any dose-rate dependent differences in normoxia (16–18). Contrary to these findings, for two murine pancreatic cancer cell lines, Venkatesulu et al. found a reversed FLASH effect at normoxic conditions (19).

Consequently, there is a need for further *in vitro* studies allowing for experiments in a controlled oxygen environment (1, 20). In the present work, we have performed a comparative study of FLASH *vs.* CONV and assessed clonogenic survival, DNA damage, and cell cycle synchronization under normoxic conditions for a range of different cell lines. Our investigations show that the FLASH effect may occur at relatively low doses under normoxic conditions and that it depends on cell-line specific variations in susceptibility.

## Material and Methods

### Cell Culture

The human breast cancer cell lines MCF7 and MDA-MB-231, the human fibroblast cell line MRC-5, and the human cervix cancer cell line HeLa (in the study two different HeLa cells were used; early passage cells and a high passage subclone) were acquired from American Type Culture Collection (ATCC). The human colon cancer cell line WiDr was acquired from LGC Promochem (Teddington, UK). The squamous cell carcinoma LU-HNSCC4 was established in our laboratory from a patient with a squamous cell carcinoma in the floor of the mouth (21). Cells were grown in monolayers in DMEM (MCF7, MDA-MB-231, WiDr, HeLa, LU-HNSCC4) or EMEM (MRC5) media with 10% fetal bovine serum and 1% penicillin-streptomycin at 37°C in a humidified atmosphere with 5% CO_2_. All cell lines were confirmed to be negative for mycoplasma infection.

### Clonogenic Assays

Exponentially growing cells were trypsinized and plated in appropriate cell densities in 2.50 ml medium per Falcon T12.5 flask (Thermo Fischer Scientific TM, Waltham, MA) and allowed to adhere overnight before irradiation. Control flasks for determination of the plating efficiency and the FLASH- and CONV-flasks were prepared identically on the same occasion, for each repetition. FLASH and CONV-flasks were irradiated minutes apart with doses from 0-9 Gy (12 Gy for HeLa_subclone_). Irradiation was performed under normoxic conditions at room temperature with the flasks lying flat and irradiated from beneath (beam angle 180 degrees). After irradiation, the flasks were returned to the incubator for 9-14 days. All flasks, including the non-irradiated controls, were terminated at the same occasion. Cells were fixed and stained with methylene-blue in 70% ethanol. Flasks were scanned using a flatbed scanner in 1,200 dpi resolution. Colony counts were performed with a standardized ImageJ-code (version 1.53e, Wayne Rasband, National Institute of Health, USA) and manually checked. Surviving fraction (SF) was determined as the number of colonies with at least 50 cells divided by the number of plated cells (corrected for plating efficiency).

### DNA-Double Strand Break Foci Formation

150 000 - 500 000 cells were plated in Slide-Flasks (Thermo Fischer Scientific Nunc, Roskilde, Denmark) and allowed to adhere overnight before irradiation with 3 Gy. At specific time points after irradiation, cells were washed with PBS and fixed with 4% paraformaldehyde for 20 min. After washing, cells were permeabilized with 0.5% Triton-X100 in PBS for 20 min, washed, blocked in blocking buffer (0.2% skimmed milk, 0.1% TritonX-100, 5% FBS in PBS) for 1 hour followed by 1 hour incubation with 53BP1 primary antibody (Invitrogen PA146147) and 1 hour of incubation with a secondary antibody (AlexaFluor anti-rabbit 488). Cell nuclei were counterstained with DAPI. Permeabilization, washing, blocking and staining steps were all performed at room temperature. Foci formation were assessed with a widefield fluorescence microscope, AxioOberver Z.1 (Zeiss, Oberkochen, Germany), equipped with ×63/1.40 Plan-Apochromat oil-immersion objective lens and Colibri 7 solid state LED light source (Zeiss), and an ORCA-Flash4.0 V3 Digital CMOS camera (Hamamatsu Photonics, Hamamatsu City, Japan). In each sample, at four different positions, fifteen Z-stack images were acquired, deconvoluted with a GPU-based deconvolution module and averaged using a Maximum Intensity Projection-algorithm (Black Zen Imaging Software, Zeiss). ImageJ was used for automated foci identification and quantification of DNA-double strand break (DSB) foci.

### Cell Cycle Analyses

150 000 - 500 000 cells were plated in 35 mm or 60 mm Petri Dishes (Corning, Corning, NY, USA) and allowed to adhere overnight before irradiation with 6 Gy (and 3 Gy for the HeLa_subclone_ cells). At 24 h (6 h and 24 h for HeLa_subclone_ cells), after irradiation cells were washed with PBS, harvested and fixated with ice-cold ethanol (70%). Cell nuclei were stained with propidium iodide (*10 lg/ml, RNase A 0.1 lg/ml*) for 30 min at room temperature and DNA content was determined with an Accuri C6 Flow Cytometer (Becton Dickinson, Franklin Lakes, NJ, USA). DNA-histograms were analyzed in ModFit LT 5.0 for Mac (BD Biosciences).

### Irradiation and Dosimetry

Irradiation and dosimetry were performed as described previously (12). In summary, a modified (22) Elekta Precise (Elekta AB, Stockholm, Sweden) medical linear accelerator (LINAC) was used for irradiations with FLASH and CONV dose rates with a 10 MeV electron beam. The average dose rate for CONV irradiation was 14 Gy/min. For FLASH irradiation, the average dose rate was ≥800 Gy/s, delivered with an integer number of 3.5 μs pulses, with a dose-per-pulse of 3.0 Gy, and a pulse repetition frequency of 200 Hz. Thus, the instantaneous pulse dose rate was 0.86 MGy/s, which is the same as the average dose rate for the 3 Gy single pulse delivery, while the average dose rate was 1.2 kGy/s for the 6 Gy delivery, 900 Gy/s for the 9 Gy delivery, and 800 Gy/s for the 12 Gy delivery. GafChromic EBT3 film (Ashland Specialty Ingredients G.P., Bridgewater, NJ) was used for dosimetry for both FLASH and CONV irradiation. Dose measurements were performed in conjunction with each cell experiment. In addition, online dose delivery verification measurements were performed. For CONV irradiation, these were performed with the built-in monitor (transmission) chamber. For FLASH irradiation, a Farmer-type ionization chamber placed at a specific position in the ceiling of the treatment room (furthest possible distance from the source) was used.

### Statistical Analyses

RStudio v. 1.0.136 (RStudio Team (2015). RStudio, Inc., Boston, MA, URL http://www.rstudio.com/) was used for statistical calculations. The parameters of the linear–quadratic model (23) (SF=exp(−αD−βD^2^)) were fitted to the log (SF) using the nonlinear least-squares method (‘nls’ in RStudio). Two alternative models were fitted, one with separate α and β parameters for the CONV and FLASH data, and one with common α and β parameters for all data. The residuals were tested for normality using the Kolmogorov-Smirnov test, and the F-test was used to determine whether the fit was significantly improved by using separate parameters. Using the model fitted with separate α and β parameters for the CONV and FLASH data, dose-modifying factors (DMF) were determined as the ratio of D_FLASH_/D_CONV_ at a survival fraction of 0.1 (SF=0.1) and 0.01 (SF=0.01). Boot-strapping was used to calculate the median and the interquartile range of the DMF. In addition, the difference in survival fraction at the individual dose levels were tested for statistical significance, without assuming normality, by using the Wilcoxon rank-sum test. All tests were two-sided with a chosen significance level of 5%. Experiments were repeated 2-4 times.

## Results

### Clonogenic Assays

**Figure 1:**
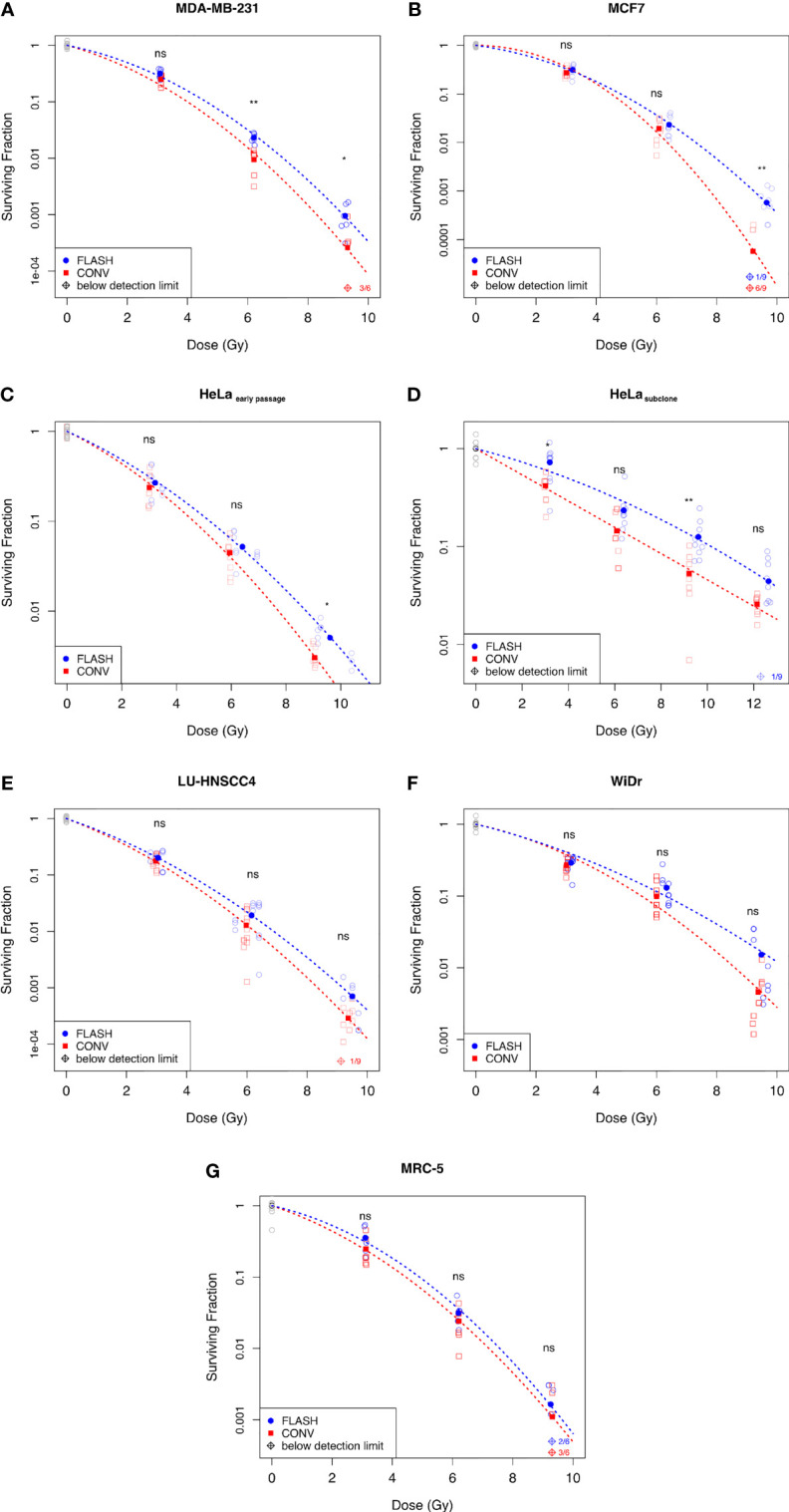
Surviving fraction assessed by clonogenic assay comparing FLASH with conventional dose rates (CONV) for human *in vitro-*cell lines; **(A)** Breast cancer cell line MDA-MB-231, **(B)** Breast cancer cell line MCF7, **(C)** Cervix cancer cell line HeLa_early passage_, **(D)** HeLa_subclone_ **(E)** Head&neck cancer cell line LU-HNSCC4, **(F)** Colon cancer cell line WiDr, and **(G)** Normal lung fibroblasts MRC-5. Blue circles denote FLASH, red squares denote CONV, and grey circles denote the non-irradiated controls. The empty symbols represent the individual flasks and the filled symbols represent the average surviving fraction at the dose indicated. The dashed lines illustrate the fitted survival curve according to the linear quadratic model. Diamond symbols denote samples below the detection limit (no surviving colonies). Statistical analyses using Wilcoxon Rank-Sum test; ns, not significant, *p < 0.05, **p < 0.01. Data from three independent experiments.

A tendency of increased survival after FLASH compared with CONV was suggested for all cell lines (**Figure 1**), with significant differences for four of the seven cell lines. A general FLASH-sparing was also indicated by a DMF at SF=0.1 of around 1.1 for all cell lines, except the HeLa_subclone,_ for which it was around 1.3 (**Table 1**). The use of separate α and β parameters for FLASH and CONV resulted in significantly improved fits for the MCF7, LU-HNSCC4, HeLa_early passage_ and HeLa_subclone_, indicating differences across the curves as a whole. Significant survival differences were also observed for MDA-MB-231 at 6 Gy and 9 Gy. The WiDr and MRC-5 cell lines did not show any significant differences in survival after FLASH compared with CONV in the dose range studied.

**Table 1:** Dose modifying factors (DMF) at a surviving fraction (SF) of 0.1 and 0.01 for the various cell lines.

Cell line	DMF (SF=0.1)	IQR (SF=0.1)	DMF (SF=0.01)	IQR (SF=0.01)	F-test *p*-value
WiDr	1.16	1.03-1.29	1.20	1.10-1.30	0.34
MCF7	1.10	1.00-1.20	1.16	1.11-1.21	0.03
LU-HNSCC4	1.12	0.94-1.30	1.15	1.06-1.24	0.007
MRC-5	1.09	0.97-1.21	1.05	1.00-1.10	0.24
MDA-MB-231	1.14	1.02-1.26	1.12	1.07-1.17	0.15
HeLa_early passage_	1.12	1.02-1.22	1.13	1.09-1.17	0.04
HeLa_subclone_	1.32	1.19-1.45	NA	NA	0.05

The F-test denotes the significance level for separate parameter sets for FLASH and CONV, compared with one common fit. IQR; inter-quartile range; NA, Not Applicable.

### DSB—Foci With 53BP1

**Figure 2:**
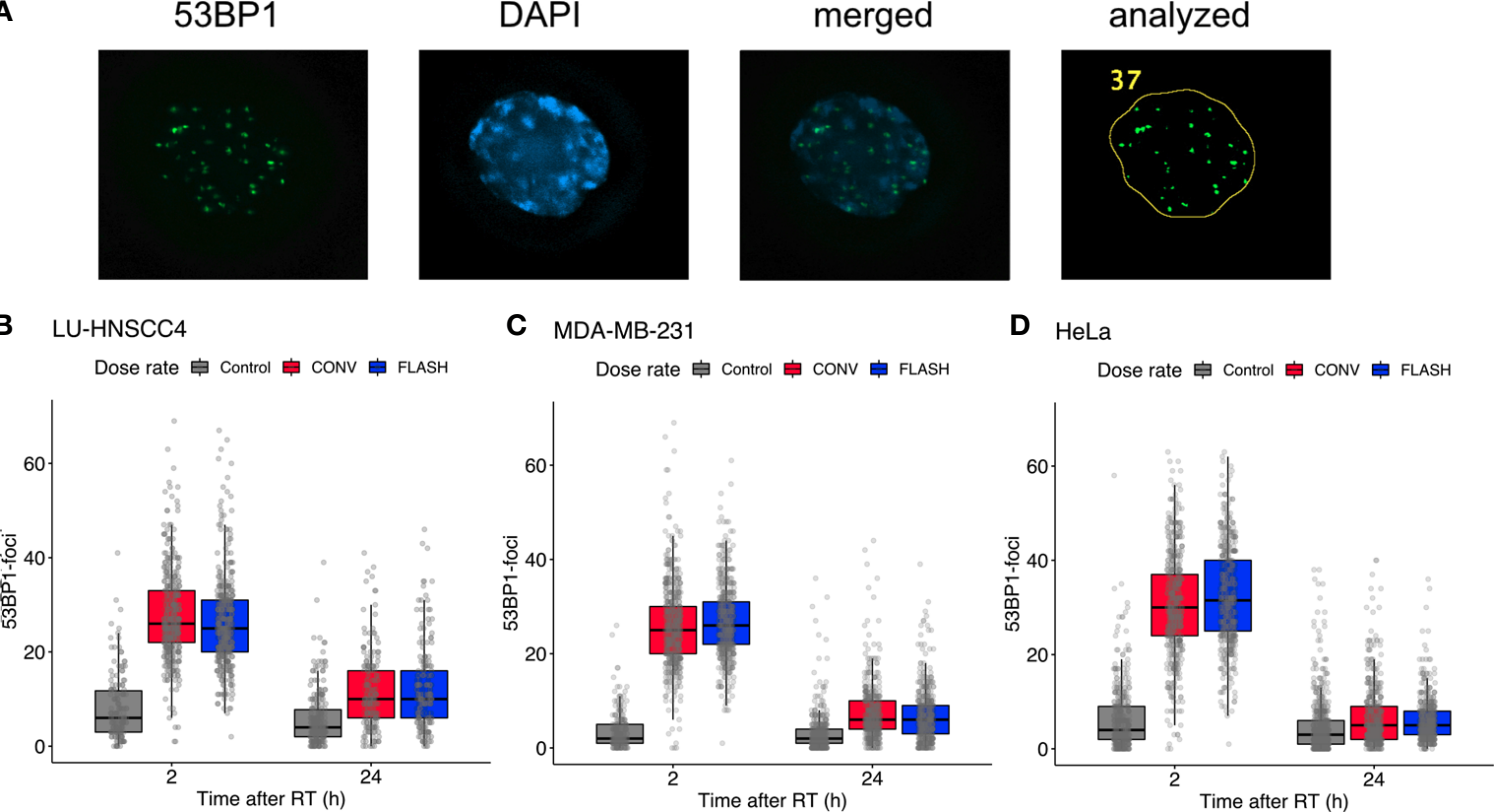
Evaluation of radiation-induced DNA-double strand break foci using 53BP1. **(A)** Representative microscopy image showing (left to right) 53BP1, DAPI, merged image, and the resulting analyzed image after processing in ImageJ. **(B–D)** Number of 53BP1 foci at 2 h and 24 h after 3 Gy irradiation with FLASH (blue) or conventional dose rate (CONV, red) compared with controls (grey) for LU-HNSCC4 (**B**; 1,532 scored cells), MDA-MB-231 (**C**; 2,583 scored cells), and HeLa_subclone_ (**D**; 2,973 scored cells). The box and whisker plots illustrate median (thick line), interquartile range (box) and the lowest/highest observation within ±1.5* interquartile range (IQR) from the box (whiskers). The individually scored cells are indicated with transparent circles. Data from two independent experiments.

Three of the cancer cell lines, LU-HNSCC4, MDA-MB-231 and HeLa_subclone_, were further studied for radiation-induced DNA-DSB using the 53BP1-marker at 2 h and 24 h after irradiation with 3 Gy (**Figure 2**). A marked induction of DNA-DSB foci was seen at 2 h after irradiation, and declined substantially at 24 h. Comparing FLASH and CONV, median foci numbers were similar with overlapping interquartile ranges, for all three studied cell lines (**Table 2**).

**Table 2:** Number of DSB-foci per cell with the 53BP1-marker for three cell lines at 2 h and 24 h after irradiation with FLASH or conventional dose rate (CONV), and for non-irradiated controls (Ctrl).

Cell line	Ctrl at 2 h	FLASH at 2 h	CONV at 2 h	Ctrl at 24 h	FLASH at 24 h	CONV at 24 h
	Median (IQR)	Median (IQR)	Median (IQR)	Median (IQR)	Median (IQR)	Median (IQR)
LU-HNSCC4	6 (3–12)	25 (20–31)	26 (22-33)	4 (2-8)	10 (6-16)	10 (6-16)
MDA-MB-231	2 (1-5)	26 (22-31)	25 (20-30)	2 (1-4)	6 (3-9)	6 (4-10)
HeLa_subclone_	4 (2-9)	32 (25-40)	30 (24-37)	3 (1-6)	5 (3-8)	5 (2-9)

IQR, Inter-quartile range.

### Cell Cycle Analyses

**Figure 3:**
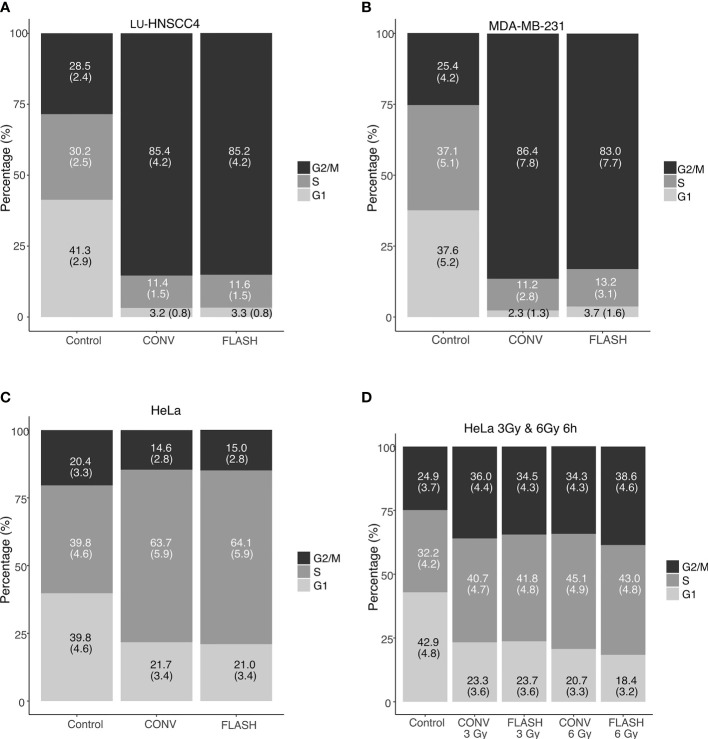
Cell cycle distributions determined by flow cytometry after irradiation with FLASH or conventional dose rate (CONV). In **(A–C)**, cell cycle distribution 24 h after irradiation with 6 Gy for LU-HNSCC4 **(A)**, MDA-MB-231 **(B)** and HeLa_subclone_ **(C)**. In **(D)** an earlier time-point (6 h) after irradiation with 3 Gy and 6 Gy using the HeLa_subclone_. Bars illustrate G1 (light grey), S-phase (grey), and G2/M (black). The figures in the bars denote the percentage of cells (with standard deviations). Data from two independent experiments.

To further investigate potential differences in radiation response between FLASH and CONV, radiation-induced cell cycle arrest was investigated for MDA-MD-231, LU-HNSCC4 and HeLa_subclone_ cells. At 24 hours after irradiation with 6 Gy, both FLASH and CONV induced cell cycle synchronizations in the three cell lines (**Figures 3A–C**). Interestingly, HeLa_subclone_ cells were predominantly synchronized in early S-phase (the S-phase was sub-analyzed in three compartments, **Supplementary Figure S1**), whereas the MDA-MB-231 and LU-HNSCC4 cells were synchronized in the G2/M-phase. To elucidate if the HeLa_subclone_ cell synchronization in early S-phase was due to a previous transient G2/M-arrest, we studied an earlier time point, 6 h after irradiation and an additional dose level, 3 Gy, and found radiation-induced G2/M-arrest (**Figure 3D**). The cell cycle analyses could not resolve any significant differences between FLASH and CONV.

## Discussion

We have found *in vitro* evidence of a FLASH sparing effect measured with clonogenic survival occurring under normoxic conditions for several cancer cell lines. The magnitude of the FLASH effect differed between the cell lines and was most pronounced for HeLa_subclone_ cells, with a significant sparing already apparent at 3 Gy. The normal lung fibroblasts did not show any significant difference in survival between FLASH and CONV. Cell cycle synchronization and DSB-foci formation were assessed for three of the cancer cell lines with similar responses for FLASH and CONV exposures.

It is well recognized that FLASH spares normal tissues *in vivo* (2–4, 13, 24). However, available *in vitro* results with clonogenic assays in normoxia are inconsistent both in recent (12–15, 19) and older studies (25–30). Consistent with our current findings, an increased survival fraction after irradiation with ultra-high dose rate in normoxia have been reported (13, 29, 30), whereas other results indicate no difference (14, 15, 25–28) or a reversed effect (19). The inconsistent results could indicate differing intrinsic biological susceptibility for FLASH. In addition, survival differences have been shown for hypoxic *in vitro-*conditions (12, 27, 28, 31, 32). In the present study, the magnitude of the FLASH effect, expressed as DMF at SF=0.1, was around 1.1 for six of the seven cell lines, while a DMF of 1.3 was found for the HeLa_subclone_. The values are in line with previously published *in vivo* data, generally showing a DMF of 1.2-1.5 (7). The HeLa_subclone_ data (with passage number approaching 40) show a distinct behavior compared to the other cell lines, with a larger DMF and also earlier cell cycle arrest. In comparison with HeLa_early passage_, the HeLa_subclone_ was considerably more radioresistant and lacked a shouldered survival curve for the CONV-irradiated samples. It has previously been reported that phenotype changes can occur with high passage numbers, affecting radiation responses (33). The present results showing differences between cell lines, together with the inconsistent findings by others, suggest that the FLASH effect might not be an independent, universal dose-modifying factor. Instead, the sparing effect could involve biological determinants varying from cell line to cell line.

DSB-foci formations were numerically in the same range for the three studied cancer cell lines, with similar foci numbers for FLASH compared with CONV irradiation. Fouillade et al., using the same DSB-marker at an earlier time point (30 minutes), showed a lower number of foci for FLASH compared with CONV for normal lung fibroblasts, but no differences for the A549 lung cancer cell line (24). Cell cycle synchronization after irradiation was seen for the three studied cell lines in the current study, but with similar effects after both FLASH and CONV irradiation. Auer et al. also studied cell cycle synchronization of HeLa cells after irradiation with 3 Gy using laser-accelerated protons at different dose rates. They found a less pronounced G2/M-accumulation at 10 h for cells irradiated with ultra-high dose rate compared with conventional dose rate, but no differences at 24 h (34). The HeLa_subclone_ cells used in the current study revealed no synchronization in G2/M-phase at 24 h after irradiation, instead the cells were synchronized in early S-phase. Additional experiments at an earlier time-point (6 h after irradiation) with two different doses (3 Gy and 6 Gy) indicated an earlier radiation induced G2/M-synchronization, suggesting a peak of the G2/M-arrest at a time point before 24 h (**Supplementary Figure S1**). However, the cell cycle synchronization was similar after FLASH and CONV at both 6 h and 24 h.

FLASH effects are typically seen at doses ≥ 10 Gy. The current study showed a separation of the survival curves at doses below 10 Gy. Interestingly, also using a low dose of 4 Gy, Chabi et al. found FLASH irradiation to be more efficient than conventional dose rate exposures for two cases of T-cell lymphoblastic leukemia (T-ALL), but an opposite relation for a third case (35). The results underpin that FLASH effects do not exclusively occur at high doses and also suggest that intrinsic biological factors might determine the FLASH response. The authors proposed that genomic profiles might predict when FLASH is beneficial. Additional investigations in the nature and mechanism of such biological determinants, and their influence on the radiochemical and biological steps of the radiation response remain to be investigated. Many of the steps are likely to be influenced by the available oxygen concentration, and we have previously shown the dependence on oxygen concentration for a FLASH effect (12). However, differences between FLASH and CONV at low doses in well-oxygenated environments, i.e. where oxygen depletion is considered to be negligible, imply that the TOD hypothesis is insufficient to account for the whole FLASH effect. We therefore deduce that the FLASH effect, in part, must be caused by other mechanisms.

FLASH radiotherapy is a promising new technique and convincing reports show its ability to protect normal tissue from radiation damage (2–5, 13, 24). Most *in vivo* experiments also suggest an iso-effective tumor control compared with CONV (2, 5, 36–38), even though some studies have found other results (19, 35). The tumor’s response to radiation in the complex *in vivo* environment is dependent not only on direct cell kill but also on inflammatory reactions and the immune system, involving surrounding connective tissues. Considering a possible inherited susceptibility for cancer cells to exhibit a FLASH effect (35), which would be detrimental for tumor control, further investigation in the differential response between tumor and normal tissue is clearly indicated. Ideally, such studies will generate models that describe tissues and tumors for which a differential response can be exploited.

The current study has some limitations. Since the FLASH irradiation was delivered with an integer number of pulses (1-4), the average dose rate varied between the different dose levels. However, the dose-per-pulse and the instantaneous/pulse dose rate were constant and the average dose rate exceeded 800 Gy/s at all dose levels. Further, we have chosen to perform a pure *in vitro* study to enable studies of different cell lines under well-controlled oxygen concentrations where the impact of biological interactions was minimized. Thereby, the results are valid under these circumstances, and their generalizability to more complex biological systems need to be further investigated. The lack of a difference in the DSB-foci induction between FLASH and CONV warrants future experiments using additional methods to assess DNA-damage and repair after irradiation. The varying results between cell lines suggest that biological determinants may affect the response, but this study alone does not identify any underlying mechanisms or predictive signatures that could be further examined in a preclinical or clinical translation.

To conclude, we have found a FLASH effect under normoxic conditions for several cell lines *in vitro*, and that the magnitude of the FLASH effect differed between the cell lines. The results indicate that the FLASH effect cannot be solely explained by TOD and that other mechanisms are involved. The nature of such possible biological susceptibilities and their dependence on oxygen concentrations will be subject to further investigations.

## Data Availability Statement

The datasets presented in this article are not readily available but the datasets generated during and/or analyzed during the current study are available from the corresponding author on reasonable request. Requests to access the datasets should be directed to gabriel.adrian@med.lu.se.

## Author Contributions

GA, CC, SM, and KP designed the study. GA, SB, EK, KB, MG, and KP conducted the experiments. GA, CC, EK, SM, SB, KP, and AW analyzed and interpreted the data GA, KP, and CC drafted the manuscript. Critical revision of the manuscript for important intellectual content – all authors. GA and CC did the statistical analyses. All authors contributed to the article and approved the submitted version.

## Funding

This work was supported by Mrs Berta Kamprad Foundation (grant no 2020-19-301), Swedish Cancer Society (grant no 20 1298), John and Augusta Persson’s Foundation and governmental research funding (ST-ALF). Funding for AW was provided by the Swedish Society for Medical Research (SSMF).

## Conflict of Interest

The authors declare that the research was conducted in the absence of any commercial or financial relationships that could be construed as a potential conflict of interest.

## Publisher’s Note

All claims expressed in this article are solely those of the authors and do not necessarily represent those of their affiliated organizations, or those of the publisher, the editors and the reviewers. Any product that may be evaluated in this article, or claim that may be made by its manufacturer, is not guaranteed or endorsed by the publisher.

## Supplementary Material

Supplementary Figure 1 Cell cycle distributions determined by flow cytometry after irradiation with FLASH or conventional dose rate (CONV) for the HeLa_subclone_ cell line 24 h after irradiation with 6 Gy. The S-phase was sub-divided into S1 (early), S2 (middle) and S3 (late). Bars illustrate G1 (light grey), S-phase (grey), and G2/M (black). The figures in the bars denote the percentage of cells (with standard deviations). Data from two independent experiments.
