# Supplementary figures and images for: Cancer Cells Can Exhibit a Sparing FLASH Effect at Low Doses Under Normoxic In Vitro-Conditions
Source: Front Oncol. 2021 Jul 29;11:686142. doi: 10.3389/fonc.2021.686142 (PMC8358772; doi:10.3389/fonc.2021.686142)

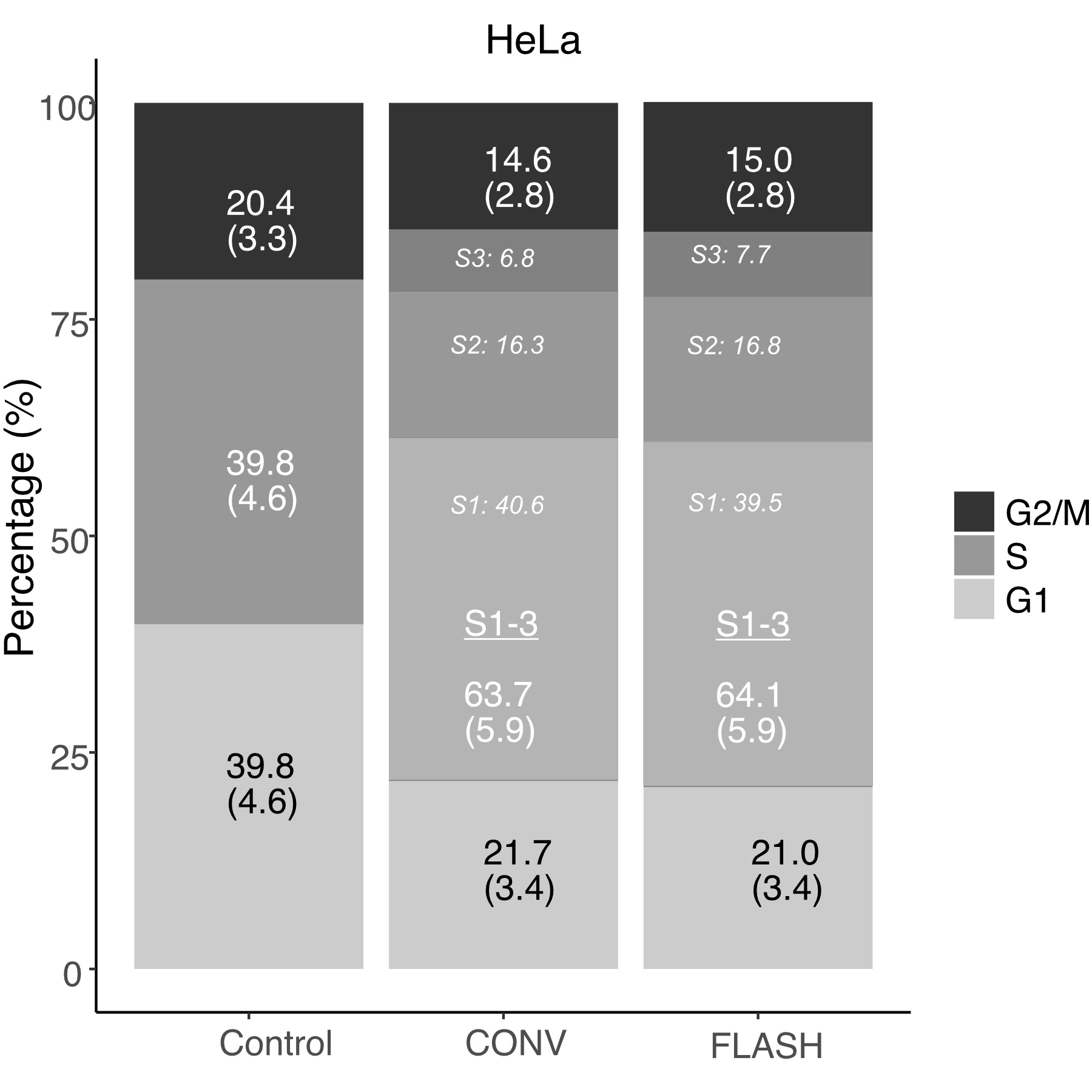

Supplement: Supplementary Figure 1 — Cell cycle distributions determined by flow cytometry after irradiation with FLASH or conventional dose rate (CONV) for the HeLasubclone cell line 24 h after irradiation with 6 Gy. The S-phase was sub-divided into S1 (early), S2 (middle) and S3 (late). Bars illustrate G1 (light grey), S-phase (grey), and G2/M (black). The figures in the bars denote the percentage of cells (with standard deviations). Data from two independent experiments. [file Image_1.tiff]
